# Renal stone density on native CT-scan as a predictor of treatment outcomes in shock wave lithotripsy

**DOI:** 10.25122/jml-2022-0153

**Published:** 2022-12

**Authors:** Samir Muter, Ziad Abd, Ruya Saeed

**Affiliations:** 1 Department of Surgery, College of Medicine, University of Baghdad, Baghdad, Iraq; 2 Department of Surgery, College of Medicine, University of Anbar, Al-Ramadi, Iraq; 3 Department of Community Medicine, College of Medicine, University of Anbar, Al-Ramadi, Iraq

**Keywords:** ESWL, native CT, stone density, renal stone, CT – Computed tomography, ESWL – Extracorporeal Shock Wave Lithotripsy, f URS – flexible ureteroscopy, HU – Hounsfield unit, KUB – Kidneys, ureters, bladder, UTI – Urinary tract infection

## Abstract

Extracorporeal shock wave lithotripsy (ESWL) is considered a standard treatment for nephrolith or kidney stones measuring less than 20 mm. Anatomical, machine-related, and stone factors play pivotal roles in treatment outcomes, the latter being the leading role. This paper examined the relationship between stone density on native CT scans and ESWL treatment to remove renal stones concerning several treatments. One hundred and twenty patients (64 males and 56 females) were enrolled and completed the study from April 2019 to September 2020. Inclusion criteria were a single renal pelvis stone of 5–20 mm to be treated for the first time in adult patients with no urinary or musculoskeletal anatomical abnormalities. We assessed patients' renal function and obtained stone characteristics using a native CT scan. Patients were then scheduled for ESWL by the same machine and operator under fluoroscopy, with two-week intervals between treatment sessions when more than one treatment session was required. Before each new session, a new KUB-US was performed to reevaluate the stone. One hundred and twenty patient records were analyzed, 64 (53.3%) males and 56 (46.7%) females, with a mean age of 38.6 years and a mean stone size of 13.15 mm. Treatment with ESWL cleared stones in 76 (63.3%) patients, while 44 (36.7%) failed the treatment. The mean stone density in patients whose stones were cleared was significantly lower (661 *vs*. 1001) (P<0.001). Estimating renal calculus (or kidney stone) density on a native CT scan might help prognosticate ESWL treatment outcomes regarding stone clearance rates and the number of sessions required to clear a stone.

## INTRODUCTION

Extracorporeal shock wave lithotripsy (ESWL) has been the primary therapeutic option for small to medium-sized renal stones [1]. Factors affecting treatment outcomes have been thoroughly studied in trials to determine the primary influence, and nomograms were created to help predict treatment outcomes [2]. In addition to the shock wave generator itself, many stone characteristics have been the core of research, including stone size, site, and composition [1, 2]. In 1988, Dretler brought the concept of stone brittleness [3], after which the stone composition became the most crucial factor in predicting ESWL treatment success. However, a big problem emerged because, in most patients, the stone composition is unknown before treatment. Many methods have been tested to determine the stone composition and fragility on ESWL, including pH, urinary crystal determination, bone densitometry, and radiological evaluation [4, 5]. Historically, stones were evaluated with plain KUB X-ray films, ultrasonography, and excretory computerized tomography urograms. Native CT scan is now regarded as the gold-standard imaging for evaluating patients with renal colic and kidney stones because it provides rapid and accurate information about the stone, with more significant density discrimination than conventional radiographs [6, 7].

Furthermore, it can distinguish between stones and other radiolucent filling defects depending on substance density [7–9], with the latter being used to determine stone composition [10, 11]. Important stone characteristics include stone size and composition. One major problem is the lack of information on the stone composition before therapy. Many studies tried to link stone density on native CT scans measured by Hounsfield units (HU) to stone composition and used it to predict treatment outcomes.

Over the past several years, flexible ureteroscopy (fURS) and intracorporeal laser lithotripsy have emerged as competitive treatment choices for small and medium-sized renal stones with high efficacy and safety profiles [12, 13]. With the pros and cons of the two treatment modalities, counseling and assisting patients with renal stones and choosing one modality over the other might be an uneasy task for the urologist. Stone density on native CT scans is a readily available and easy-to-measure feature that might be used to provide success rate estimates to patients as accurately as possible [13, 14].

This paper assessed the role of stone density on native CT in prognosticating stone-free rates after ESWL therapy of renal stones.

## MATERIAL AND METHODS

One hundred and fifty-seven patients with solitary renal pelvis stones scheduled for ESWL at Al-Ramadi Teaching Hospital consented and enrolled in this prospective observational study between April 2019 to September 2020. Still, only one hundred and twenty patients (64 males and 56 females) completed the study and were considered for statistical analysis. Inclusion criteria were adults with a solitary renal pelvis stone treated for the first time, stone size between 5 and 20 mm, and no urinary or skeletal anatomical abnormalities. Patients with the same previous side or current ureteric stents were excluded. Moreover, patients with increased body mass index (BMI >30) were also excluded from the study.

### Study design

First, all patients were assessed, and medical history and physical examination were performed. Then, a complete list of laboratory investigations was ordered according to the hospital protocols. These include thorough blood count, renal and liver function tests, and screening tests to check the coagulation profile and urinalysis. Urine culture was ordered only for patients with evidence of urinary tract infection (UTI) on simple urinalysis, and all patients with active UTI were excluded from the study.

All patients had a native CT scan at diagnosis or later if their stones were visualized first by ultrasonography. According to the hospital protocols, it is a standard practice for all patients scheduled for ESWL to have native CT scans (Philips brilliance 64 slice model 2006 with 3 mm slice thickness section, 120 kV, 300 MA).

In this research, we focused on stone density in CT scans and did not consider the stone shape. However, we also considered the stone size, an important parameter in response to SWL.

Sections were taken through the renal calculi to determine the stone dimensions and density utilizing soft tissue settings of a window width level of 360 and 60 Hounsfield units, individually. A multiplanar reconstruction (MPR) was then performed for more accurate stone characterization. The mean density of each stone was measured in both axial and MPR images in four sites, and the mean was calculated and used in this study. CT interpretation and stone density measurement was carried out by a uroradiologist with more than 5 years of experience. All patients were treated by ESWL Piezolith 3000 plus 2017 (Richard Wolf) under sedation by an expert operator with over 10 years of experience. Stone fragmentation was monitored by fluoroscopy. The hospital ESWL treatment protocol starts with 0.1 KV and increases gradually stepwise to a maximum of 4.0 KV. In each ESWL session, a maximum of 4000 shock waves were delivered at a frequency of 1.5 Hz. Two-week intervals were kept between retreatment sessions. Before each new session, an ultrasound of the kidney, ureter and bladder (KUB-US) was performed to re-assess the stone. A stone of less than 5 mm was considered clinically insignificant residual fragments (CIRF) and was treated conservatively, and the case counted as a success. A maximum of 4 treatment sessions were offered. Patients who failed the 4^th^ session were referred back to the urology outpatient department to discuss other possible treatment options and were counted as a failure.

### Data analysis

Only patients who completed the study were included in the statistical analysis. Patients lost during follow-up or who developed active UTIs that prevented or delayed further ESWL sessions or required ureteric stenting were excluded from the analytical examination.

Data were examined using the Statistical Package for Social Sciences (SPSS v.28) software, using Chi-square, two-tailed t-test, and multinominal logistic regression. A 95% confidence interval was used, and a P-value ≤0.05 was considered significant.

## RESULTS

We analyzed the records of 120 patients, including 64 (53.3%) males and 56 (46.7%) females. Their ages ranged between 19 and 70 years (mean age 38.6 years). The overall mean stone size was 13.15 mm (5–20 mm), with a non-significant difference between the responders and non-responders (12.44±3.41 *vs*. 12.1±3.12) (P>0.05). Stones were almost equally distributed between the right and left sides. 61 (51.7%) stones were on the right side, and 59 (48.3%) were on the left. Eight patients (10.5%) had stone clearance after 2 ESWL sessions, 66 (86.8%) after 3 sessions, and two patients (2.6%) after 4 sessions. No patient was stone-free after one session (Table 1).

**Table 1:** Demographic and clinical data of patients.

Analyzed parameters		No	%
**Age (years), Mean±SD (Range)**		38.6±13.8	(19–69)
**Gender**	Male	64	53.3
	Female	56	46.7
**Stone side**	Right	61	51.7
	Left	59	48.3
**Stone size (mm), Mean±SD (Range)**		13.15±4.9	(5–20)
**CT scan stone density (HFU), Mean±SD (Range)**		786.0±206.8	(290–1300)
**ESWL Response**	Yes	76	63.3
	No	44	36.7
**Number of sessions**	2	8	10.5
	3	66	86.8
	4	2	2.6

Following ESWL therapy, 76 (63.3%) patients had stone clearance, while 44 (36.7%) patients failed to respond by the fourth session. There was a significant statistical difference between the mean stone density in the responders (661±139 HU) and the non-responders (1001±98 HU) (P<0.001), as shown in Table 2. A statistically significant overall correct prediction of treatment outcome of 86.8% (P<0.001) was achieved with multinominal logistic regression, as detailed in Table 3.

**Table 2:** Demographic and clinical data of patients according to ESWL response.

Parameters		Responder		Nonresponder		P-value
		No	%	No	%	
**Age (years)**	Mean±SD (Range)	40.6±13.6 (19–69)		42.2±14.1 (20–69)		0.534
**Gender**	Male	43	56.6	21	47.7	0.349
	Female	33	43.4	23	52.3	
**Stone side**	Right	39	51.3	22	50.0	0.631
	Left	37	48.7	22	50.0	
**Stone size (mm)**	Mean±SD (Range)	12.44±3.41 (5–20)		12.1±3.1 (5–20)		0.472
**CT scan stone density (HFU)**	<400 HFU	7	9.2	-	-	0.0001*
	400	2	2.6	-	-	
	500	5	6.6	-	-	
	600	29	38.2	-	-	
	700	28	36.8	-	-	
	800	3	3.9	4	9.1	
	900	2	2.6	29	65.9	
	≥1000 HFU	-	-	11	25.0	
	Mean±SD (Range)	661±139 (290–910)		1001±98 (850–1300)		0.0001^#^

* – Significant difference between percentages using Pearson Chi-square test (X^2^-test) at 0.05 level. ^#^ – Significant difference between two independent means using Student's t-test at 0.05 level.

**Table 3:** The predictive value of stone density on response to ESWL.

Classification					
Observed	Predicted				
	No response	Response-2 sessions	Response-3 sessions	Response-4 sessions	Percent correct
**Not response**	43	0	1	0	97.7%
**Response-2**	0	8	0	0	100.0%
**Response-3**	2	1	63	0	95.5%
**Response-4**	0	0	2	0	0.0%
**Overall percentage**	37.5%	7.5%	55.0%	0.0%	95.0%

Furthermore, the number of sessions required to clear a stone significantly depended on the stone density (P=0.001) (Table 4).

**Table 4:** Correlations between the number of sessions and the stone density.

Parameter estimates									
No of sessions ^a^		B	Std. Error	Wald	df	Sig.	Exp(B) (Odd ratio)	95% Confidence Interval for Exp(B)	
								Lower bound	Upper bound
**Two sessions**	Intercept	59.991	16.020	14.024	1	.000	-	-	-
	Stone Size	.047	.249	.035	1	.852	1.048	.644	1.705
	Stone density (HFU)	-.083	.019	18.339	1	.000	.920	.886	.956
**Three sessions**	Intercept	50.566	15.595	10.513	1	.001	-	-	-
	Stone Size	-.065	.185	.124	1	.725	.937	.652	1.347
	Stone density (HFU)	-.058	.017	11.301	1	.001	.944	.913	.976
**Four sessions**	Intercept	24.752	14.076	3.092	1	.079	-	-	-
	Stone Size	.084	.208	.162	1	.687	1.087	.723	1.634
	Stone density (HFU)	-.031	.015	4.263	1	.039	.969	.941	.998

a – The reference category is: no response.

**Figure 1:**
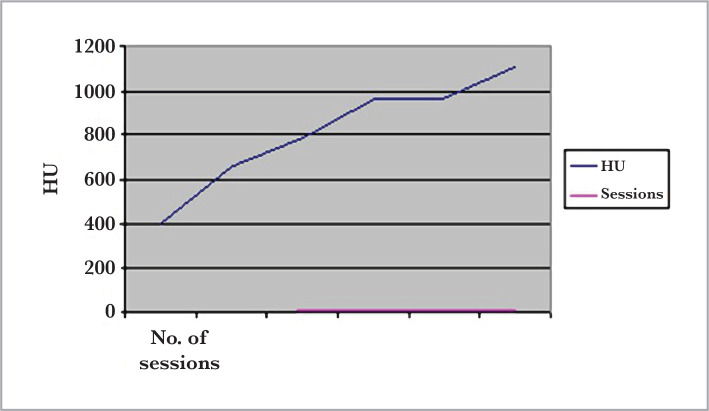
Relationship between the stone density (HU) and the number of ESWL sessions.

The number of sessions needed to clear the stone and the density (HU) had a linear relationship, as seen in Figure 1. The one-way ANOVA analysis showed significant differences in distribution (P<0.001).

Comparing the role of stone size and density on treatment outcome, the univariate analysis showed stone density to be a more excellent predictor of response and several treatments. Moreover, only stone density was a significant factor when performing stepwise logistic regression using stone density and size as independent variables and the number of sessions needed to clear a stone as the dependent variable. This is also apparent when comparing the significant predictive value (P-value) of stone size (Table 4) and stone density (Table 3). Additionally, a linear relationship was found between the stone density and the number of essential treatments for renal stones. Univariate analysis revealed that stone density is a better predictor of response and the number of sessions needed than stone size.

## DISCUSSION

Many urology guidelines consider ESWL one of the best treatment modalities for small and medium-sized renal stones [15]. Stone clearance rates and complication profiles that measure the outcomes of any treatment modality depend on many factors. These factors are related to the stone, the patient, and the technology used [2]. Stone composition is essential to predicting stone fragility, but unfortunately, it is a piece of missing information in most cases unless the patient previously had a certain type of stone, especially those with underlying metabolic derangement.

In the presence of other minimally invasive treatment options like flexible ureterorenoscopy (fURS) and laser lithotripsy, predicting ESWL success rates becomes essential in counseling patients with renal stones and helping them favor one technique over the other [15].

Different maneuvers can be used to predict the stone composition. The type of particles and crystals excreted in urine after ESWL can be used [16], while Cohen and Parkhouse tried urinalysis with scanning electron microscopy [17]. A plain abdominal X-ray is one of the initial imaging techniques used for patients with stone disease. The ability to predict stone fragility on plain X-ray films was studied very early. Chaussy and Fuchs believed that if the stone has a lesser radiodensity than the spine, it is likely to break readily on ESWL, while stones with more radiodensity than the spine are more challenging to fragment [18]. Some data showed that smooth-edged homogenous stones required more shock waves to fragment than stones with round, radially reticulated, speculated edges and irregular margins [3, 4]. Density measurement on plain X-ray is subjective and regarded as qualitative rather than quantitative, limiting its clinical use.

On the other hand, CT scan is now available, non-invasive, and can provide excellent density discrimination and quantitative measurement of stone density. In addition, the stone density measured in HU may be used to predict the stone composition and response to ESWL therapy [10, 15]. Masuomy et al. showed that the overall sensitivity of CT-scout in testing the density of urinary stones is 86.27% (from 80.8 to 86.5%) [19]. Moreover, the specificity of the CT-scout density in detecting stone consistency was 64.29%. The size of the stone can influence the sensitivity: a concernment diameter of 5 mm or more raises the sensitivity of the scanning [19].

Our study correlated stone HU obtained on native CT with its fragility. Our results revealed a negative relationship between the two; the higher the stone HU, the lesser the chance of achieving clear stone status. Therapeutic consideration can be inferred from these results by predicting the likelihood of success of ESWL therapy for renal stones, the number of sessions required for complete stone fragmentation, and the need for early consideration of other therapeutic options.

Motley et al. reported that HU determination on native CT did not predict stone composition [11]. They failed to find any significant difference in mean stone density (in HU) among calcium, uric acid, struvite, and cystine stones. A possible explanation may be the low number of uric acid stones (only 7) or the heterogenicity of stones in their sample. On the other hand, Gyan and colleagues found that although there was no significant difference in stone density when comparing calcium oxalate with calcium phosphate stone, a significant difference was observed between these two stone types and uric acid stones. This may be due to the sample's higher number of uric acid stones. They also correlated stone density with clearance rates and concluded that 36% of patients with residual calculi had a mean stone density of ≥900 HU compared to ≤500 HU in 74% of cleared stones [20]. Similarly, Newhouse et al. used native CT to measure stone density to analyze stone composition accurately. They reported that uric acid and cystine calculi could be identified, but calcium-containing calculi such as brushite and oxalate could not be differentiated from each other [21]. Mostafavi et al. (1998) [10] and Michael et al. (2010) [22] used dual-energy CT scans and could determine stone composition more precisely. They could even differentiate between different types of calcium-containing stones, such as brushite and oxalate from struvite stones.

Saw et al. [23] investigated the relationship between the calculus attenuation value in an *in vitro* analysis, and several shock waves were required to break it into pieces. They concluded that generally, for calcium stones, the number of shock waves needed to fragment a stone was less than half its attenuation value (HU); this is what they called the "half-attenuation rule", which predicted the number of shock waves needed to break 95% of cases in their study. In an interesting *in vivo* study, Nakada et al. [24] studied the attenuation size ratio (peak attenuation/calculus size) concerning the findings from the calculus analysis. They found a significant difference between uric acid stones, with a mean of 344 HU, and calcium oxalate stones, at 652 HU. By utilizing an attenuation/size ratio threshold of >80, the negative predictive value that the calculus would be predominantly made of calcium oxalate was 99%.

It was observed by Joseph et al. that patients with calculi of <500 HU had complete clearance and required 2500 shock waves, whereas those patients with calculi of 500–1000 HU had a clearance rate of 86% and needed 3390 shock waves. Lastly, patients with calculi of ≥1000 HU had a clearance rate of 55%, demanding 7300 shock waves. They recommended that percutaneous nephrolithotomy should be considered if the attenuation value of the calculus was >950 HU and 7500 shock waves did not achieve adequate fragmentation [25].

This prospective observational study investigated the relationship between stone density and size with treatment outcomes. Higher stone density was associated with lower clearance rates and more treatment sessions. Furthermore, stone density was a better predictor of ESWL success than stone size itself. This study also opened the prospect for possible treatment of larger stones (>2 cm) with low stone density using ESWL.

## CONCLUSION

Our findings suggest that a pretreatment measurement of stone density (in HU) using a native CT scan might be a good predictor of stone-free rates following ESWL treatment of small and medium-sized renal stones. Moreover, it is a better predictor of treatment outcome than stone size. This information could be used for counseling patients with renal stones when choosing between different treatment modalities, especially ESWL and retrograde intrarenal surgery in the era of flexible ureteroscopy. We recommend measuring stone density for all patients scheduled for ESWL therapy before counseling and commencing treatment, which will increase overall patient satisfaction and reduce the retreatment burden on the health system.

## Data Availability

Further data is available from the corresponding author on reasonable request.

## References

[1] Herout R, Baunacke M, Groeben C, Aksoy C (2022). Contemporary treatment trends for upper urinary tract stones in a total population analysis in Germany from 2006 to 2019: will shock wave lithotripsy become extinct?. World J Urol.

[2] Iqbal N, Hassan A, Singh G, Hassan MH (2021). use of computed tomography-based nomogram in adult age patients to predict success rates after shock wave lithotripsy for renal stones: a single center experience. J Ayub Med Coll.

[3] Fan J, Zhang T, Zhu W, Gurioli A (2019). The role of super-mini percutaneous nephrolithotomy (SMP) in the treatment of symptomatic lower pole renal stones (LPSs) after the failure of shockwave lithotripsy (SWL) or retrograde intrarenal surgery (RIRS). Urolithiasis.

[4] Dretler SP, Polykoff G (1996). Calcium oxalate stone morphology: fine tuning our therapeutic distinctions. J Urol.

[5] Jacobsen MC, Thrower SL, Ger RB, Leng S (2020). Multi-energy computed tomography and material quantification: Current barriers and opportunities for advancement. Medical Physics.

[6] Fielding JR, Steele G, Fox A, Heller H, Loughlin KR (1997). Spiral computerized tomography in the evaluation of acute flank pain: a replacement for excretory urography. J Urol.

[7] Lalchan S, Sharma P, Subash KC, Gyawali M, Poudel A (2022). Diagnostic Accuracy of Ultrasonography in Detecting Ureteric Calculi in Patients with Renal Colic Taking Non-Contrast Multidetector Computerized Tomography of Kidney, Ureter, and Bladder (CT KUB) as the Gold Standard. Nepal J Med Sci.

[8] Wibulpolprasert P, Jungtheerapanich S, Wibulpolprasert B (2019). Distinguishing infiltrative transitional cell carcinoma from other infiltrative lesions of the kidneys on multidetector computed tomography. Ramathibodi Med J.

[9] Masoumi N, Langroudi TF, Bagheri F, Alirezaei A (2021). Determining the opacity of urinary stone using only the Computed Tomography imaging, Is KUB still needed?. Men's Health J.

[10] Mostafavi MR, Ernst RD, Saltzman B (1998). Accurate determination of chemical composition of urinary calculi by spiral computerized tomography. J Urol.

[11] Motley G, Dalrymple N, Keesling C, Fischer J, Harmon W (2001). Hounsfield unit density in the determination of urinary stone composition. Urology.

[12] Geraghty RM, Jones P, Somani BK (2017). Worldwide trends of urinary stone disease treatment over the last two decades: a systematic review. J Endourol.

[13] Hsieh TY, Chen SL, Chang YR, Tyan YS, Chen TR (2022). Effective dose for kidney-ureter-bladder plain radiography, intravenous urography, and abdominal computed tomography scan: A phantom study. Applied Radiation and Isotopes.

[14] Choo MS, Uhmn S, Kim JK, Han JH (2018). A prediction model using machine learning algorithm for assessing stone-free status after single session shock wave lithotripsy to treat ureteral stones. J Urol.

[15] Skolarikos A, Jung HU, Neisius A (2022). Guidelines on Nephrolithiasis. Eu Associat Urol (Arnhem, Netherlands).

[16] Pozdzik A, Maalouf N, Letavernier E, Brocheriou I (2019). Meeting report of the "Symposium on kidney stones and mineral metabolism: calcium kidney stones in 2017". J Nephrol.

[17] Cohen NP, Parkhouse H, Scott ML, Bowsher WG (1992). Prediction or response to lithotripsy: the use of scanning electron microscopy and X-ray energy dispersive spectroscopy. Br J Urol.

[18] Chaussy CG, Fuchs GJ (1989). Current state and future developments of non-invasive treatment of human urinary stones with extracorporeal shock wave lithotripsy. J Urol.

[19] Masoumi N, Langroudi TF, Bagheri F, Alirezaei A (2021). Determining the opacity of urinary stone using only the Computed Tomography imaging, Is KUB still needed?. Men's Health J.

[20] Pareek G, Armenakas NA, Fracchia JA (2003). Hounsfield units on computerized tomography predict stone-free rates after extracorporeal shock wave lithotripsy. J Urol.

[21] Newhouse JH, Prien EL, Amis JrES, Dretler SP, Pfister RC (1984). Computed tomographic analysis of urinary calculi. Am J Roentgenol.

[22] Ferrandino MN, Boll DT, Pierre SA, Patil NA (2009). Dual Energy Computed Tomography With Advanced Post-Image Acquisition Data Processing: Improved *In Vitro* And *In Vivo* Determination Of Urinary Stone Composition. J Urol.

[23] Saw KC, McAteer JA, Fineberg NS, Monga AG (2000). Calcium stone fragility is predicted by helical CT attenuation values. J Endourol.

[24] Nakada SY, Hoff DG, Attai S, Heisey D (2000). Determination of stone composition by noncontrast spiral computed tomography in the clinical setting. Urology.

[25] Joseph P, Mandal AK, Singh SK, Mandal P (2002). Computerized tomography attenuation value of renal calculus: can it predict successful fragmentation of the calculus by extracorporeal shock wave lithotripsy? A preliminary study. J Urol.

